# Temporal frequency of events rather than speed dilates perceived duration of moving objects

**DOI:** 10.1038/srep08825

**Published:** 2015-03-06

**Authors:** Daniel Linares, Andrei Gorea

**Affiliations:** 1 University of Barcelona, Faculty of Psychology, Basic Psychology Department, Barcelona, Spain; 2 Laboratoire Psychologie de la Perception, Université Paris Descartes, Paris, France; 3 Centre National de la Recherche Scientifique, Paris, France

## Abstract

In everyday life moving objects often follow irregular or repetitive trajectories for which distinctive events are potentially noticeable. It is known that the perceived duration of moving objects is distorted, but whether the distortion is due to the temporal frequency of the events or to the speed of the objects remains unclear. Disentangling the contribution of these factors to perceived duration distortions is ecologically relevant: if perceived duration were dependent on speed, it should contract with the distance from the observer to the moving objects. Here, we asked observers to estimate the perceived duration of an object rotating at different speeds and radii and found that perceived duration dilated with temporal frequency of rotations, rather than speed (or perceived speed, which we also measured). We also found that the dilation was larger for two than for one object, but the increase was not large enough to make perceived duration independent of the number of objects when expressed as a function of the local frequency (the number of times an object crossed a given location per time unit). These results suggest that perceived duration of natural stimuli containing distinctive events doesn't depend on the distance of the events to the observer.

The perceived duration of intervals of hundreds of milliseconds to a few seconds—a time scale in which many daily live actions unfold—is distorted by the dynamics of visual stimulation^1^,^2^,^3^,^4^,^5^,^6^,^7^,^8^,^9^,^10^,^11^,^12^,^13^,^14^,^15^. It is unclear, however, which aspect of the dynamics is critical in perceived duration distortions and hence, which are the underpinning mechanisms and possible function of such distortions. When the dynamic stimulus contains distinctive events—such as changes in contrast (e.g. a flickering blob)— perceived duration dilates with the *temporal frequency* of the events^2^,^5^ up to about 4–8 Hz^5^ when the dilation saturates or even starts decreasing^12^. Sometimes, however, dynamic stimuli do not contain distinctive events, while perceived duration is nevertheless distorted. For an object moving along a rectilinear trajectory, for example, perceived duration dilates with its *speed*^5^,^16^.

In everyday life moving objects, such as the body parts of an animal, often follow irregular or repetitive trajectories for which distinctive events—like direction changes or oscillations—are potentially noticeable. Perceived duration dilates for objects moving along such irregular^4^ or repetitive trajectories^1^,^3^. Whether the dilation is due to the *(perceived) temporal frequency* or to the *(perceived)* *speed* remains unclear. Kanai et al^5^. and Kaneko and Murakami^7^ addressed this question using moving gratings of different spatial and temporal frequencies. Kanai et al.'s results suggest that temporal frequency rather than speed is the main factor inducing dilations, but Kaneko and Murakami's results suggest that it is speed. The reasons of the discrepancy are unclear. Furthermore, it is unknown whether the perceived duration of moving gratings—in which the motion of the internal texture is inconsistent with the overall displacement of the stimulus —and of more usual translating objects is subject to equivalent distortions. In spatial vision, for example, biases in spatial localization of moving gratings and of translating objects have different characteristics^17^. Similar dissociations might occur for duration perception. Hence the debate on whether the critical factor dilating perceived duration is temporal frequency or speed remains open, particularly so for the case of translating objects.

Disentangling the contribution of temporal frequency and speed to perceived duration distortions is ecologically relevant. Objects moving with the same speed in the environment yield different retinal speeds with their distance from the observer so that objects moving far away yield lower speeds on the retina and should therefore be perceived as lasting less than objects moving close by (e.g. Gorea and Hau^18^). At the same time, the temporal frequency of events—such as the number of oscillations per time unit of a waving hand—is distance independent.

Assuming that any salient change constitutes an event that can result in distortions of perceived duration, one must consider that in dynamic stimuli such events usually occur simultaneously at different temporal rates and at different spatial scales^19^. A minimal example consists of two objects rotating along the same circular trajectory. Each location on the trajectory is crossed with a temporal frequency (hereafter referred to as *local* frequency) twice the temporal frequency of the rotations (hereafter referred to as *global* frequency). Whether such local or global (possibly more cognitive) “events” are the critical factors potentially inducing perceived duration distortions is no less an open question than the one concerning the contribution to such distortions of speed versus temporal frequency. The present study pits each of these three factors against each other.

Observers had to estimate the duration of appearance of one or two luminance blobs rotating about fixation on a circular trajectory at different speeds and radii (Fig. 1). If perceived duration depends on speed, it should be independent of the radius of the trajectory and of the number of blobs. If it depends on rotational (i.e. global) frequency, it should be insensitive to the number of blobs and should change with the radius of the trajectory in such a way that the perceived duration of objects moving at different radii should be the same as long as the rotational frequency is the same. Finally, if perceived duration depends on local frequency, it should be sensitive to both the radius and the number of blobs with two rotating blobs inducing the same duration distortion as one blob rotating at twice the speed.

## Results

### Rotational frequency vs. speed

For observers to extract the rotational frequency of the stimulus, the blobs might need to cover a relatively large angular distance. The trajectory of the blobs would otherwise be too similar to a rectilinear trajectory for observers to be able to determine the number of laps or fractions of a lap. This suggests that in order to pit rotational frequency against speed, relatively fast rotational frequencies and long durations should be used. Nonetheless, in order to compare our results with those of previous studies^3^,^4^,^5^,^7^,^16^, we included in our experiments two duration ranges (means 0.939 s and 1.871 s) and a wide range of speeds including slow speeds. We expected that for short durations and slow speeds, rotational frequency could not be the critical factor modulating perceived duration because observers wouldn't be able to compute it.

Figure 2A shows the normalized perceived duration of one moving blob (perceived duration divided by the average duration; see Data analysis) averaged across observers (geometric mean) as a function of speed for the two radii (plotted in different colors) and for the two (short and long) average durations (plotted in the top and bottom graphs respectively). Consistent with previous findings^3^,^4^,^5^,^7^,^16^, perceived duration increases with speed (about 30% of dilation in our case) over a wide range of speeds. For the slowest speeds (3.98 and 6.31 deg/s for the short average duration and 3.98 deg/s for the long average duration range) perceived duration is independent of the radius of the trajectory (in this figure and in all the figures to be discussed below, asterisks indicate significance levels; see legends and Data analysis). For the remaining, much larger range of speeds, perceived duration is significantly longer for the 2-deg-radius than for 4-deg-radius trajectory. This indicates that, within this large range of speeds, speed per se cannot account for the modulation of perceived duration.

Figure 2C shows the averaged normalized perceived duration this time as a function of rotational frequency. As expected, for short durations and slow rotational frequencies (approximately bellow 0.7 rps), rotational frequency is not the critical factor modulating perceived duration: perceived duration is larger for the 4-deg-radius than for the 2-deg-radius trajectory. For the remaining conditions, perceived duration does not depend on the radius (at our significance levels), consistent with rotational frequency being the critical factor inducing the perceived duration dilation.

With one exception the pattern of the results for two moving blobs (Fig. 2B and 2D) is quite similar to that for one blob. The exception is observed for the short average duration and the slowest speed for which the perceived duration of the two blobs configuration does depend on the radius of the trajectory (top graph in Fig. 2B).

Recently it has been shown that duration perception depends on *perceived* rather than *physical* speed^20^. As perceived speed is known to decrease with eccentricity^21^,^22^,^23^,^24^, it is possible that, when plotted against rotational frequency, the observed drop in perceived duration with the radius of the circular trajectory was in fact due to a decrease in perceived speed. To test this possibility, we assessed perceived speed by asking 3 of the 7 observers to match the speeds of the stimuli used in the duration experiment with the speed of an object moving on a rectilinear trajectory (see Methods).

Figure 3 shows each observer's speed matching data with radius (3A) and number of blobs (3B) as parameters. Perceived speed does not depend on the number of rotating blobs (1 or 2; strong overlap of the confidence intervals in Fig. 3B) but, consistent with previous findings, it decreases with the radius (2 and 4 deg; non-overlap of the confidence intervals in Fig. 3A). Consequently, the observed difference in perceived duration between the 2 and 4 deg radii (Fig. 2A) is smaller when plotted against perceived speed (Fig. 4B) than when plotted against physical speed (Fig. 4A). Nonetheless, consistent with the averaged data displayed in Figure 2, the individual data displayed in Figure 4 sustain the observation that rotational frequency is the critical factor inducing the perceived duration dilation: the radius effect on perceived duration disappears when the latter is plotted as a function of rotational frequency (Fig. 4C).

We also assessed perceived rotational frequency by asking the remaining 4 of the 7 observers to match the rotational frequency of a blob moving on a circular trajectory of radius 3 deg to the rotational frequencies of our stimuli (radii 2 and 4 deg). Observers MC and SY were able to match rotational frequency for different radii (Fig. 5A) and number of blobs (Fig. 5B), but observers FE and AB were not (Fig. 5A and 5B). For most rotational frequencies the rotational frequency perceived by FE and AB increases with the radius suggesting that they were matching speed rather than rotational frequency (Fig. 5A). This difficulty to estimate rotational speed has been signaled in previous studies^25^,^26^. For the observers who were able to match rotational frequency (MC and SY), perceived rotational frequency makes perceived duration less dependent on the radii than physical rotational frequency, but with only two observers it is hard to draw strong conclusions (Fig. 6).

### Rotational frequency vs. local frequency

Figure 7 shows the data from Figure 2 plotted this time as a function of rotational (Fig. 7A and 7B) and local frequency (rotational frequency multiplied by the number of blobs; Fig. 7C and 7D) with the number of blobs (plotted in different colors) and the two (short and long) average durations (plotted in the top and bottom graphs respectively) as parameters. With the exception of the highest rotational frequencies around 2 rps, perceived duration as a function of the rotational frequency is longer for two blobs than for one blob (Fig. 7A and 7B). When plotted as a function of the local frequency, perceived duration is shorter for two blobs than for one blob (Fig. 7C and 7D). The two observations taken together indicate that rotational or local frequency alone cannot account for the perceived duration dilation independently of the number of blobs; the number of blobs also needs to be taken into account.

## Discussion

We found that for a wide range of speeds, the perceived duration of a rotating object increased with the rotational frequency independently of the radius of its trajectory. This suggests that for objects moving along trajectories for which distinctive events—like rotations—are noticeable, the temporal frequency of these events rather than object's speed is the critical factor modulating perceived duration. By extension, the present results also suggest that the perceived duration of natural stimuli containing distinctive events, such as the waving hand of a person, should be independent of the events' distance from the observer.

For two rotating objects perceived duration also increased with the rotational frequency independently of the radius of the trajectory, but the overall perceived duration was larger for two objects than for one object. This increase in perceived duration with the *number* of rotating objects, however, was not large enough to make *local frequency* (the number of times an object crossed a given location per unit of time) the critical factor modulating perceived duration. These results indicate that neither rotational frequency nor local frequency can explain by themselves the distortions caused by the number of objects.

For short durations and slow rotational frequencies, rotational frequency did not account for observers' perceived duration: for the same rotational frequency perceived duration depended on the radius of the trajectory. This result was expected given that for short durations and slow rotational frequencies the trajectory covered by the objects is difficult to discriminate from a rectilinear trajectory thereby preventing observers from extracting the number of laps or fractions of a lap per unit of time. Under these conditions of non-compelling perceived rotation, we found that *speed* was the critical factor modulating perceived duration: perceived duration increased with speed independently of the radius of the trajectory.

### Effect of speed on perceived duration

The modulation of perceived duration with speed for quasi-linear trajectories that we found for short durations and slow rotational frequencies is consistent with previous measurements of perceived duration for stimuli moving at a constant speed^7^,^16^. Under such conditions, it could be the covered distance instead of speed per se that modulates duration (the Kappa effect^27^). It is however unlikely that distance play a significant role in the perceived duration dilations observed with stimuli translating within a limited spatial window such as random-dot kinematograms^5^,^28^ for which spatial crowding of their component elements (dots) impair the perception of their individual trajectories.

The dependence of perceived duration on speed that Kaneko and Murakami^7^ found for moving gratings could be accounted for, as they suggested, by the fact that the temporal changes in their stimuli—like in our stimuli for short durations and slow rotational frequencies—were not salient enough for extracting their temporal frequency.

It has been hypothesized that perceived duration increases with the strength of the neural activity in response to a stimulus^29^,^30^. Consequently, one possibility is that that speed expands perceived duration because it increases neural response^20^. Consistent with this idea, the amplitude of the EEG^31^, MEG^32^ and fMRI signals increases with speed^33^,^34^ (up to some moderate speeds).

### Effect of number of objects on perceived duration

The presently found increase in perceived duration with the number of objects is consistent with Xuan et al.'s^35^ results showing that the perceived duration for 8 or 9 static objects was larger than for 1 or 2 objects, but is not consistent with Brown's^4^ finding that the perceived durations of 1, 3 or 5 moving objects do not differ. This inconsistency remains to be explained.

Given that in our displays the total size and the luminous flux are larger for two than for one object, it is possible that these low level factors instead of the number of objects account for the duration dilation. As there is some evidence that neural response increase with numerosity, size and luminance^29^, the increases in perceived duration that we obtained for two objects relative to one object is consistent with the proposal that perceived duration increases with the strength of the evoked neural response.

### Effect of temporal frequency on perceived duration

The increase in perceived duration with rotational or local frequency independently of the radius of the trajectory—our main finding—is tantamount with a *decrease* for a given speed of perceived duration with the radius of the trajectory. As this dependency was not observed for objects rotating at low speeds (i.e. not displaying a compelling rotational motion), it is unlikely that the decrease in perceived duration is due to eccentricity as shown to be the case for static objects^36^,^37^.

*Perceived speed* is known to decreases with eccentricity^21^,^22^,^23^,^24^, a finding that we replicated. The decrease in perceived speed with eccentricity, however, was not large enough to make perceived speed the critical factor modulating perceived duration.

As discussed above, it is difficult to decide whether, for objects moving along rectilinear trajectories, speed or distance is the critical perceived duration modulating factor. In a similar vein, it is difficult to know whether the presently found dependence of perceived duration on rotational frequency could not be instead accounted for by the total angular distance travelled by the rotating object.

Consistent with our results, Kanai et al^5^. found that for moving gratings temporal frequency was the critical factor modulating perceived duration. According to Kaneko and Murakami^7^, Kanai et al.'s results were the consequence of a discernable overall luminance modulation of their stimuli causing a discernible flicker percept. However, such global flicker was present only for the lowest spatial frequency gratings in Kanai et al.'s study, while their reported duration dilation was observed for both low and high spatial frequencies (see their Experiment 4). Given that Kanai et al. used expanding concentric gratings centered on the fixation point, it is possible that the luminance changes around the fovea were salient enough to make temporal frequency the important factor. Our results indicate that perceptually distinctive events, even if they are not associated with overall luminance changes, make temporal frequency the critical factor modulating perceived duration.

We found evidence of non-monotonic increases in perceived duration with temporal frequency. First, for observer AD perceived duration increased with frequency for fast frequencies, but decreased with frequency for slow frequencies. Such a U-shape function was also evident in the data of the authors of this paper obtained in pilot experiments and in the results of a previous study^3^. Second, although our data averaged across observers do not show a saturation of perceived duration with frequency, individual data of most of our observers do show such evidence. The lack of saturation for the averaged data is consistent with previous results obtained with flickering blobs showing saturation only for temporal frequencies larger than 4–8 Hz^5^. The non-monotonic perceived duration functions of speed suggest that duration distortions are not the consequence of a response bias induced by the increase in the magnitude of some stimulus attribute^38^,^39^ such as speed or temporal frequency.

Consistent with the hypothesis that perceived duration increases with the strength of the neural response up to some moderate local temporal frequencies, the amplitude of EEG^40^,^41^, MEG^42^ and fMRI^43^ signals increases with frequency. For higher frequencies, the amplitude of the neural response saturates and then decreases with frequency^40^,^41^,^42^,^43^. These findings are in accord with the observed saturation^5^ and decrease^12^ of perceived duration at higher frequencies. Whether the neural response also increases with rotational frequency, or more generally with the temporal frequency of global (possibly more cognitive) events, is not known^29^.

In short, our results indicate that the perceived duration of a given interval containing perceptually distinctive (local or global) events increases with their temporal frequency, which suggests that the perceived duration of objects in everyday life that follow trajectories for which distinctive events are noticeable does not depend on the distance of the events to the observer. For moving objects in the absence of such distinctive events, perceived duration depends on their speed or, alternatively, on their covered distance. In general our results are consistent with the proposal that perceived duration is enhanced in proportion with the neural activity evoked by the events to be timed. A more definite support to this proposal will be to show that neural activity increases with rotational frequency independently of the radius of the trajectory.

## Methods

### Observers

Ten naïve observers were originally recruited. After being informed on the stimuli to be presented and on their task, they provided written consent to perform the experiments. Three observers were dropped: one because she failed to show at the lab after the first day of data collection and two because an analysis of their first blocks of trials suggested that they confounded duration with speed. The seven remaining observers participated first in the duration experiment, and then in the speed and frequency matching experiments. Because observers had limited available time for running the matching experiments, three were run in the speed matching experiment and the remaining four in the frequency matching experiment. This study was conducted in accordance with the requirements of the Helsinki convention and approved by the local ethical committee of Université Paris Descartes.

### Stimuli

Stimuli were generated using PsychoPy^44^. They were displayed on a large screen (Samsung Smart TV; 120 cm width × 68 cm height; 1920 × 1080 pixels; 120 Hz refresh rate), and viewed from a distance of 120 cm in a dimly lit room.

**Figure 1:**
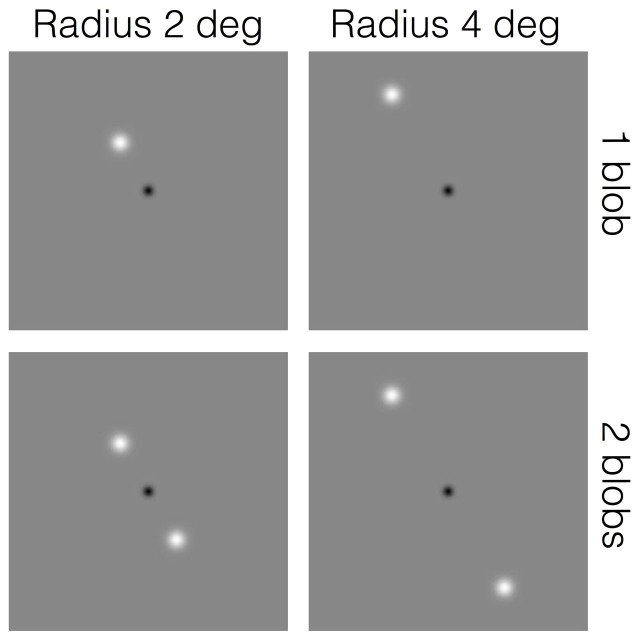
Schematic illustration of the stimuli used in the duration estimation experiment.

The stimuli (Fig. 1) consisted of one or two white Gaussian blobs of standard deviation, SD, 0.167 deg of visual angle and peak luminance of 107 cd/m^2^ (the diameter of visibility was about 1 deg) moving around a black Gaussian fixation blob (SD: 0.167 deg; peak luminance: 0 cd/m^2^) at a constant speed. They were presented against a uniform grey background (luminance: 75 cd/m^2^).

### Procedure

The black fixation blob at the center of the screen was always present (Fig. 1). Observers were instructed to fixate it during the experiments.

#### Duration estimation experiment

Observers initiated the first trial by pressing a button of the mouse. After a random interval (0.5 to 1.5 s), the moving blob(s) was (were) displayed for a duration chosen from a range of five logarithmically spaced durations. Observers were instructed to report whether the displayed duration was shorter or longer than the average duration across all previous trials of the block by pressing one of two keys of the keyboard (method of single stimuli^45^). The key-press initiated the next trial. To have an initial idea of the average duration to be displayed, observers were first run over about 20 practice trials. The duration of the stimuli, the direction of rotation (clockwise or counter-clockwise), the number of blobs (one or two), the radius of the circular trajectory (2 and 4 deg) and the speed of the blobs (3.98, 6.31, 10.00, 15.85, 25.12 and 39.18 deg/s) were randomly chosen across trials.

Two duration ranges were run in independent blocks of trials. For the *long duration* range the durations were geometrically centered on 1.871 s (1.416, 1.633, 1.867, 2.150 and 2.467 s). For the *short duration* range, they were geometrically centered on 0.939 s (0.717, .817, .933, 1.083 and 1.233 s for all but two observers – MS and SY – for who wider ranges were chosen as an analysis of their first blocks of trials revealed that the original durations did not span a sufficient range of their derived psychometric functions).

#### Speed matching experiment

Observers were presented with a *test* stimulus that they could replace any time by pressing one of two keys with a *match* stimulus. The test stimulus could be any of the stimuli described above (i.e. one or two blobs rotating along trajectories of one of two radii in one of two directions and at one of six speeds). The match stimulus was a blob identical to the test blobs, but moving at a constant speed along a 6 deg horizontal trajectory centered on fixation, but 3 deg above or below it.

Observers were instructed to adjust the speed of the match stimulus by using the mouse wheel until they believed it matched the speed of the rotating test blob or blobs. The initial speed of the match stimulus on each trial was 0.1 deg/s (very low) or 60 deg/s (very high), chosen at random. Observers were encouraged to switch between the test and match stimuli as many times as they believed necessary. Once satisfied with their adjustment, they pressed the mouse button to initiate the next trial.

Within one trial, the initial direction of both the test and match stimuli was randomly chosen, but changed to the opposite direction every 1.867 s (with an interval of 0.5 s between presentations).

#### Rotational (global) frequency matching experiment

The procedure was the same as the one for the speed matching experiment, but this time the match stimulus moved at a constant speed also on a circular trajectory of radius 3° (i.e. at half distance between the radii of the test stimuli). Its initial direction was also chosen at random, but changed to a new random direction (clockwise or counter-clockwise) every 1.867 s (with an interval of 0.5 s between presentations).

Observers were instructed to modify the speed of the match until they believed it equaled the *rotational frequency* of the test stimulus. The initial speed on each trial was 0.1 deg/s or 60 deg/s, chosen at random. The experimenter insisted that a frequency match was realized when the test and match stimuli appeared to complete a full lap within the same duration.

#### Blocks and number of trials

For the duration estimation experiment, participants AB, FE, MA and MP collected data first for long duration stimuli and then for short duration stimuli. Participants AD, MC and SY collected data in the opposite order. Each observer performed 6 blocks of trials for each average duration completing 5760 trials in total (2 average durations × 5 testing durations × 2 directions × 2 number of blobs × 2 radii × 6 speeds × 4 trials per block × 6 blocks = 5760). Each observer completed up to 6 blocks per day over an average total of 4 days distributed over 3 to 6 days.

For the speed and frequency matching experiments for each of the 2 blob numbers, 2 radii and 6 speeds each observer completed 6 matches (144 trials total) excepting observers AD and MP who collected only 5 (120 trials total) and 4 (96 trials total), respectively.

### Data analysis

#### Duration estimation experiment

For each observer, number of blobs, radius of the trajectory, speed and average duration of the stimuli (geometric mean of 0.939 or 1.871 s), we fitted the proportion of trials for which the observer responded ‘longer than average' as a function of the duration of the stimulus with a cumulative normal psychometric function estimated by maximum likelihood^46^,^47^. Forty-eight psychometric functions were thus fitted for each observer.

For each psychometric function we estimated the Point of Subjective Equality (PSE) as the duration of the stimulus for which the proportion indicated by the psychometric function was 50%. For the block of trials with an average duration of 0.939 s, a PSE of 0.8 s, for example, would indicate that 0.8 s interval was perceived as lasting 0.939 s indicating a perceived duration *dilation* of 0.139 s. This is because a PSE smaller than the actual average duration is equivalent to a leftward shift of the psychometric function meaning that observers respond more often ‘longer than average'. Consequently, the perceived duration of the average duration across the block would be 1.078 s (0.939 s + 0.139 s = 1.078 s). More generally, for each observer, average duration and condition we calculated the *perceived duration* as the *average duration + (average duration – PSE)*. We then calculated the *normalized perceived duration* as *perceived duration / average duration*.

To calculate the confidence intervals of the perceived duration for each condition, we generated for each observer a sample of perceived durations using parametric bootstrap^46^,^47^, and then, geometrically averaged the samples across observers to obtain one average sample. We repeated this procedure a 1000 times to obtain 1000 average samples and used the 2.5% and 97.5% percentiles as the confidence intervals.

**Figure 2:**
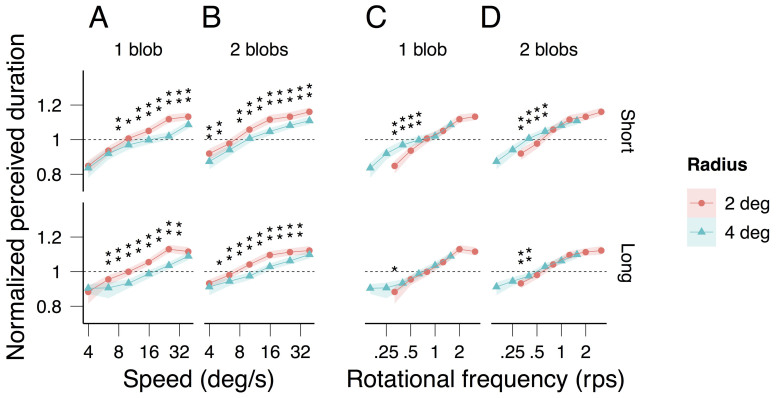
Averaged normalized perceived duration as a function of speed (A, B) and rotational frequency (C, D) for one (A, C) and two blobs (B, D), and for radius of 2 (red) and 4 deg (blue). The light colored areas surrounding the data points indicate 95% bootstrap confidence intervals (see Data analysis). For each speed and rotational frequency, the statistical significant differences in perceived duration for the two radii are indicated by asterisks (*: 95% significance level, **: 99% significance level; see Data analysis).

**Figure 3:**
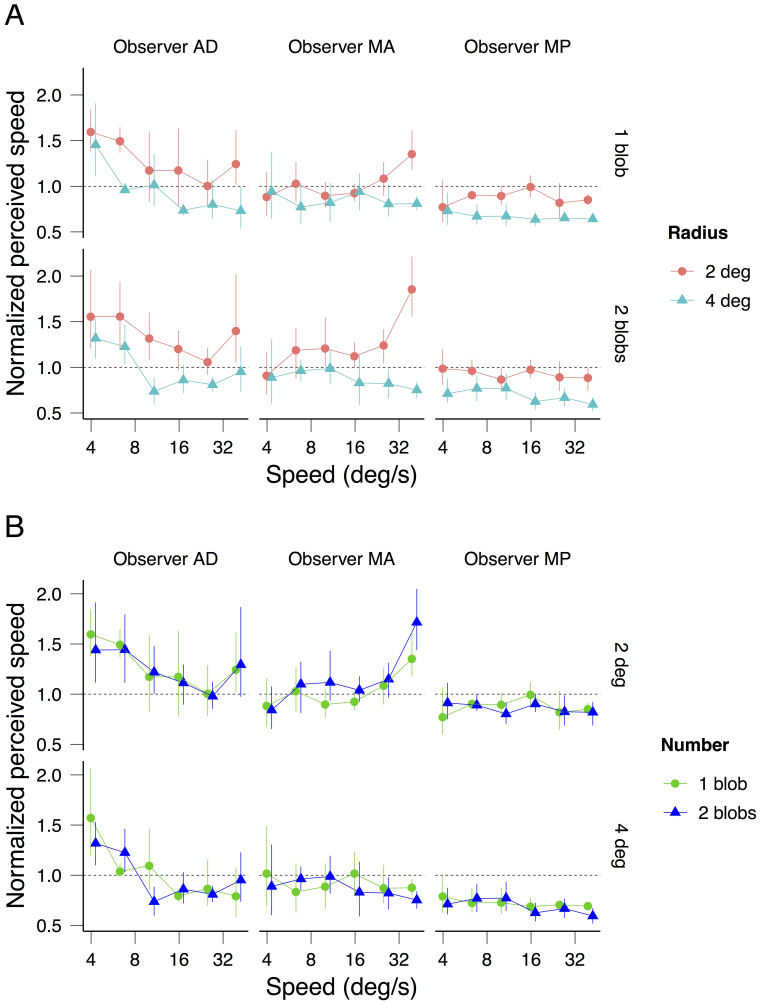
Normalized perceived speed as a function of speed for 3 observers. In (A) the results for one and two blobs are plotted on the top and the bottom graphs respectively, and the results for the different radii are plotted in different colors. In (B) the results for radius 2 and 4 deg are plotted on the top and the bottom graphs respectively, and the results for the different number of blobs are shown in different colors. The errors bars indicate the 95% bootstrap confidence intervals. To better visualize the overlap of the confidence intervals, a small spatial offset between conditions is introduced in the x-axis.

**Figure 4:**
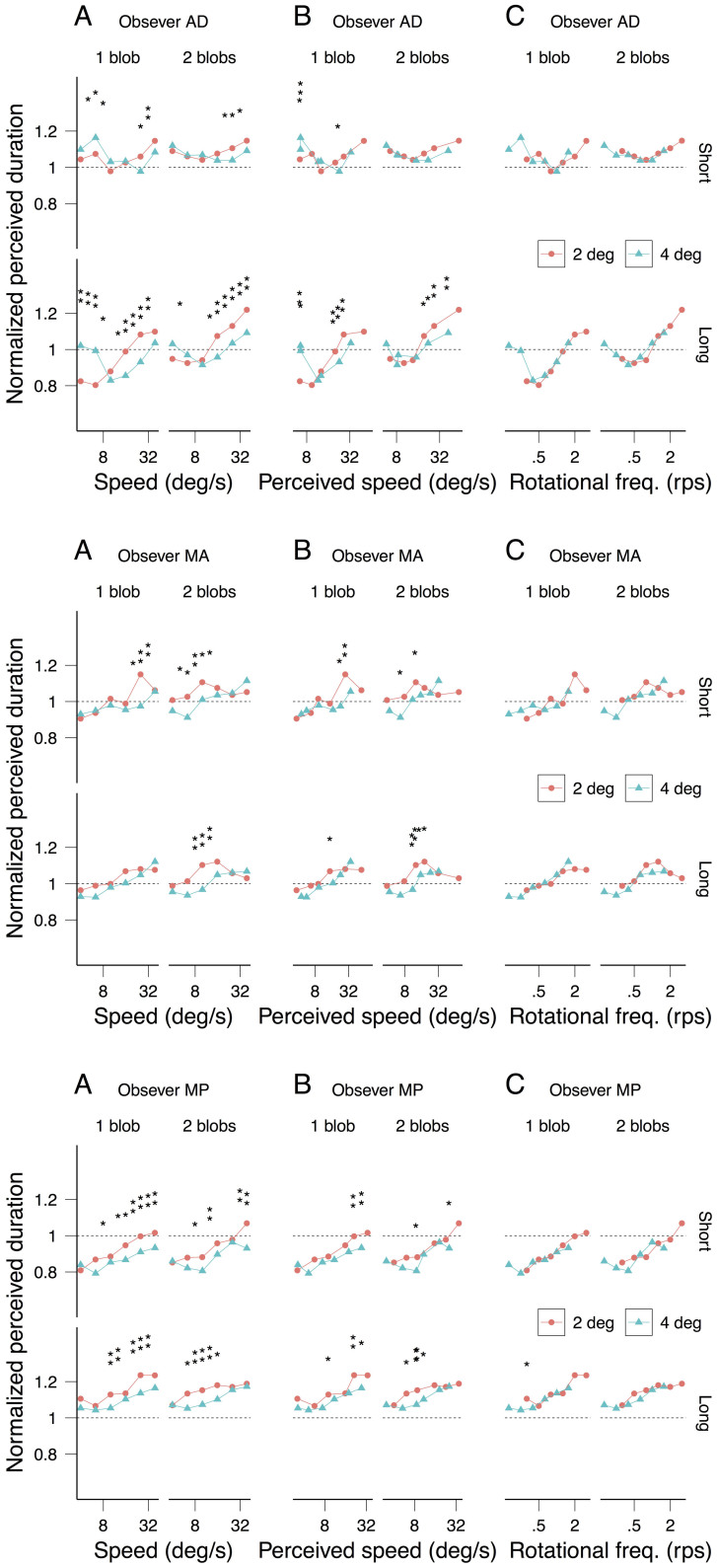
Normalized perceived duration as a function of speed (A), perceived speed (B) and rotational frequency (C) for the observers that performed the speed matching experiment (Figure 3). For each speed and rotational frequency, the statistical significant differences in perceived duration for the two radii are indicated by asterisks (*: 95% significance level, **: 99% significance level; see Data analysis).

**Figure 5:**
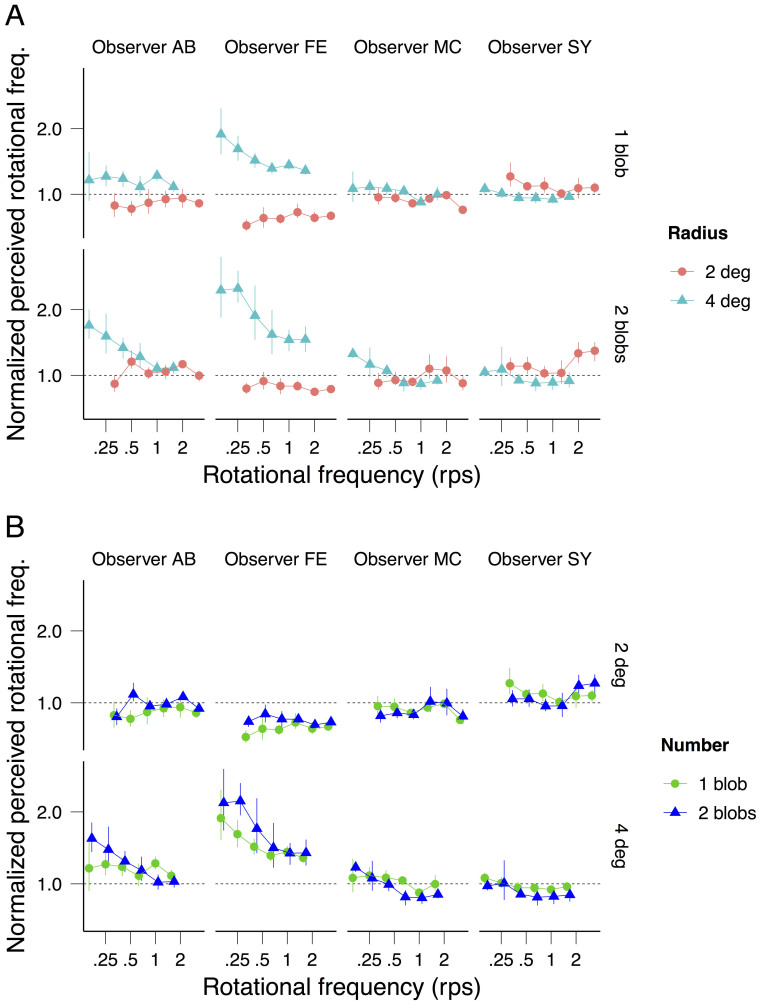
Normalized perceived rotational frequency as a function of rotational frequency for 4 observers. In (A) the results for one and two blobs are plotted on the top and the bottom graphs respectively, and the results for the different radii are plotted in different colors. In (B) the results for radius 2 and 4 deg are plotted on the top and the bottom graphs respectively, and the results for the different number of blobs are shown in different colors. The error bars indicate the 95% bootstrap confidence intervals. To better visualize the overlap of the confidence intervals, a small spatial offset between conditions is introduced on the x-axis.

**Figure 6:**
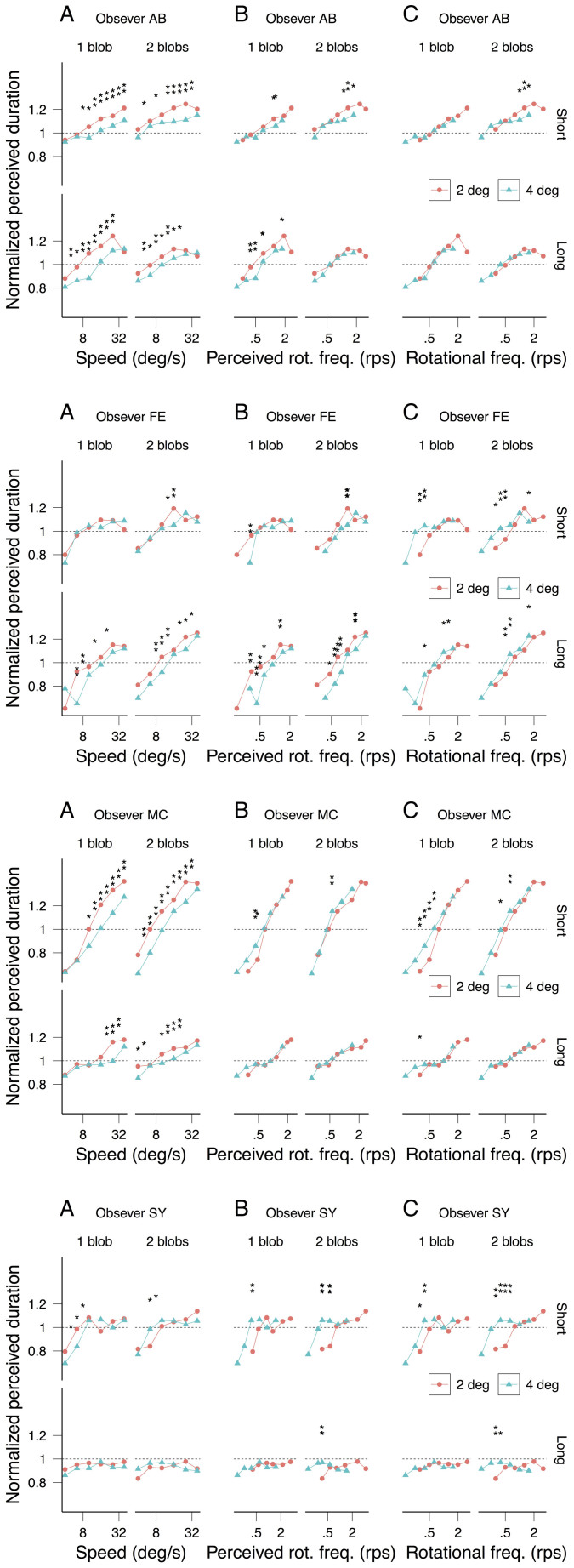
Normalized perceived duration as a function of speed (A), perceived rotational frequency (B) and rotational frequency (C) for the observers who performed the rotational frequency matching experiment (Figure 5). For each speed and rotational frequency, the statistical significant differences in perceived duration for the two radii are indicated by asterisks (*: 95% significance level, **: 99% significance level; see Data analysis).

**Figure 7:**
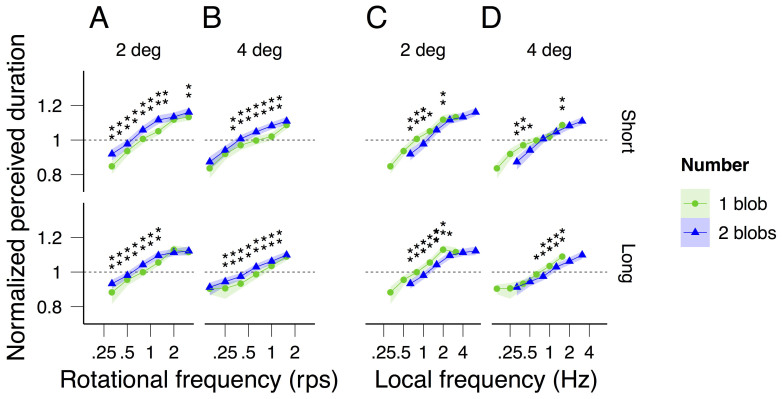
Averaged normalized perceived duration as a function of rotational frequency (A, B) and local temporal frequency (C, D) for a radius of 2 (A, C) and 4 deg (B, D), and for one (green) and two blobs (blue). The light colored areas surrounding the data points indicate the 95% bootstrap confidence intervals. For each rotational and local frequency, the statistical significant differences in perceived duration for the number-of-blobs condition are indicated by asterisks (*: 95% significance level, **: 99% significance level).

To assess whether perceived duration for a given speed differed significantly between the two radii, we generated for each observer using parametric bootstrap a sample of perceived duration for radius 2 and a sample of perceived duration for radius 4 deg, calculated the difference between the samples, and then, averaged the differences across observers to obtain one average difference. We repeated this procedure a 1000 times to obtain 1000 average differences. We considered that the two conditions were different if the null difference was within the 2.5% and 97.5% percentiles or the 0.05% and 99.5% percentiles of the distribution of the average differences (95% and 99% confidence levels respectively). The significance of the perceived duration difference between the two radii was assessed not only for the 6 speeds used in the experiment, but also for 5 speed values centered (on log axis) around these 6 speeds. We estimated perceived duration for these additional speeds by linear interpolation. The reason for using interpolated values was that for the 6 originals speeds, the rotational frequency values calculated as *speed / (2 π radius)* were not the same for radius 2 and 4 deg (Fig. 2C and 2D). Using interpolated values allowed us to assess whether the normalized perceived duration for a given rotational frequency was statistically different for the two radii. This was achieved by means of the same bootstrap procedure as for speed but now drawing bootstrap samples from the original and from the interpolated values. We also used interpolated values to assess whether perceived duration for a given rotational or local frequency (*rotational frequency x number of blobs*) differed significantly between one and two blobs (Fig. 7) and to assess whether perceived duration for a given *perceived* speed (Fig. 4) or rotational frequency (Fig. 6) differed significantly between the two radii.

#### Speed and rotational frequency matching experiments

For each observer, number of blobs, radius of the trajectory and speed, we calculated the normalized perceived speed as the geometric mean of the speed matches divided by the speed. Before averaging, we removed three speed matches of observer AD and one of observer MA because they corresponded to the speeds presented at the beginning of each matching trial (0.1 or 60 deg/s)— suggesting that observers finalized the trial without performing the match.

For each observer, number of blobs, radius of the trajectory and speed, we calculated the normalized perceived rotational frequency as the geometric mean of the rotational frequency matches divided by the rotational frequency. Before averaging, we removed two rotational frequency matches of observer SY because they were clear outliers (3.981 deg/s was matched to 630 deg/s and 39.182 to 0.3 deg/s).

## Author Contributions

D.L. and A.G. designed the study, analyzed the data and wrote the manuscript.
